# Dr. Samuel Metcalfe

**Published:** 1857-05

**Authors:** 


### NECROLOGY.

THE LATE DR. SAMUEL METCALFE.—The death of this distinguished medical philosopher merits more than a brief notice in our columns, and we greatly regret that we cannot enter more fully into the details of his life.

Dr. Metcalfe was born in Frederick County, Virginia, Sept. 21st, 1798, but his parents removed to Kentucky during his childhood, where he spent the first years of his life. Of an ardent temperament, with high aspirations, and an intense desire to distinguish himself, his thoughts and inclinations turned to a medical life; and before he obtained the wish of his heart, he had to surmount many difficulties and obstacles.

Immediately after receiving his diploma, he went to Mississippi, where he practised with success.

Whilst engaged in practice, he had his attention directed to the subject of *Caloric* as the *Vital Principle;* a theory which took firm possession of his mind; and for the more perfect study of which, he left his native country, in 1836, in order to avail himself of the resources offered to the student by the English libraries.

Before leaving, he published a volume on Terrestrial Magnetism, inscribed to Joseph Delafield, Esq., then President of the New York Lyceum of Natural History. He also contributed largely to the Knickerbocker Magazine.

He was elected to the professorship of chemistry in the Transylvania University, which his prolonged absence obliged him to resign. His work on Caloric was first published in London, in 1843, by William Pickering, and excited considerable attention amongst medical and scientific men.

About this time the Gregorian chair in the University of Edinburgh was vacated, and it was intimated to Dr. Metcalfe, that if he would become a candidate, his election was certain; but here, as on other occasions, his great modesty prevented him from seeking a situation which would, doubtless, have secured him a high position in the scientific world as a teacher.

His health failing in consequence of his unremitting mental toil, he returned to his native country, in 1845, and shortly afterwards was offered a professorship in a Southern college. But his thoughts were absorbed in his work, which he intended to republish in this country, and for which he had actually made arrangements with a publishing house in Philadelphia.

From that time up to the period of his decease, he continued working and working at his favorite theory, until his always delicate and overtasked constitution entirely gave way. He died completely exhausted on July 17th, 1856; the sad event being undoubtedly hastened by an inflammation of the lungs, he had contracted in the previous March, and from which he rallied with difficulty.

Dr. Metcalfe was a man of excellent acquirements, and an enthusiast in science, especially on the subject of Caloric.

It is much to be regretted that he possessed, in an uncommon degree, that sensitiveness which so frequently accompanies superior mental qualities. His delicate organization shrank from collision with the world, and was thus the bar to his own advancement in life.

Shut up in his study, Dr. Metcalfe endeavored to elaborate that theory which, he believed, would one day revolutionize medical science. His untimely decease did not permit its completion, and thus frustrated what had been the hope of his life. He died a martyr to his love of Natural Philosophy.

